# Pragmatism and the Importance of Interdisciplinary Teams in Investigating Personality Changes Following DBS

**DOI:** 10.1007/s12152-019-09418-3

**Published:** 2019-07-16

**Authors:** Cynthia S. Kubu, Paul J. Ford, Joshua A. Wilt, Amanda R. Merner, Michelle Montpetite, Jaclyn Zeigler, Eric Racine

**Affiliations:** 1 Center for Neurological Restoration, Cleveland Clinic, P57, 9500 Euclid Ave, Cleveland, OH 44195 USA; 2 Cleveland Clinic Lerner College of Medicine of Case Western Reserve University, Cleveland, USA; 3 Department of Psychological Sciences, Case Western Reserve University, Cleveland, USA; 4 Institut de recherches cliniques de Montréal, Montreal, Canada

**Keywords:** Deep brain stimulation, Personality, Parkinson’s disease, Ethics, Pragmatism

## Abstract

Gilbert and colleagues (2018) point out the discrepancy between the limited empirical data illustrating changes in personality (and related concepts of identity, agency, authenticity, autonomy, and self, i.e., PIAAAS) following implantation of deep brain stimulating (DBS) electrodes and the vast number of conceptual neuroethics papers implying that these changes are widespread, deleterious, and clinically significant. Their findings are reminiscent of C. P. Snow’s essay on the divide between the two cultures of the humanities (representing the conceptual publications) and the sciences (representing the empirical work). This division in the literature raises significant ethical concerns surrounding unjustified fear of personality changes in the context of DBS and negative perceptions of clinician-scientists engaged in DBS. These concerns have real world implications for funding future innovative, DBS trials aimed to reduce suffering as well as hampering true interdisciplinary scholarship. We argue that the philosophical tradition of pragmatism and the value it places on empirical inquiry, experiential knowledge, and inter-disciplinary scholarship – reflecting diverse ways of knowing – provides a framework to start to address the important questions Gilbert and colleagues raise. In particular, we highlight the importance of expert clinician knowledge in contributing to the neuroethical questions raised by Gilbert and colleagues. Finally, we provide illustrative examples of some of our interdisciplinary empirical research that demonstrate the iterative cycle of inquiry characteristic of pragmatism in which conceptual neuroethics questions have led to empirical studies whose results then raise additional conceptual questions that give rise to new empirical studies in a way that highlights the contributions of the humanities and the sciences.

## Introduction

In 1959 during the annual Rede lecture at Cambridge University, C. P. Snow made an observation that remains relevant today: namely, the divide between the two cultures of the humanities and the sciences. [1] Gilbert and colleagues’ paper [2] on the challenging question of personality changes associated with deep brain stimulation (DBS) illustrates this divide. As Gilbert and colleagues document there are an overwhelming number of conceptual neuroethics publications raising concerns that DBS can fundamentally change a patient’s personality, identity, agency, authenticity, autonomy, and self (PIAAAS) in contrast to the empirical data in which few publications demonstrate such changes (i.e., only 21/64 papers identified personality changes following DBS and only 8 of the 21 were judged to provide “significant evidence”). The disconnection between the conceptual publications and the empirical research argues for the need for greater interdisciplinary team research in this area that includes clinicians who work directly with patients and their families. Such an interdisciplinary research collaboration will ultimately result in better clinical neuroethics scholarship that, in turn, will benefit patients and society while helping inform philosophical and conceptual work in this area.

Support for our argument can be found in the philosophical tradition of pragmatism which, notably, stresses that the scientific method can be a useful model for ethical inquiries. Indeed, once a problematic situation arises, ethics represents – for pragmatists – an open-ended inquiry to search for the best possible courses of action in light of the foreseeable implications of different courses of actions. Within such inquiries, various bodies of knowledge can make significant contributions to shed light on the nature of the situation and the implications of different response-scenarios. For example, with respect to personality changes associated with DBS, knowledge about clinical aspects of patients involved, the scientific and technological features of DBS, the social and organizational context in which patients undergo DBS, etc. are all important to understand whether personality changes occur following DBS and what is the significance of those potential changes in terms of the preferable courses of action given the other options available to patients. Undertaking such inquiries builds on interdisciplinary scholarship but also reflects a commitment to incorporate experiential knowledge (i.e., different perspectives of stakeholders) and the impact of the context on moral agents involved in the situation. Engagement in open-ended or democratic deliberation about moral matters operationalizes the open-ended search for best responses using different bodies of knowledge. [3–6] As Gilbert and colleagues point out, there is a dire need for more evidence – and more critical assessment of evidence – regarding the significance of PIAAAS-related changes following DBS.

Below we establish a common frame of reference regarding personality, personality assessment, and the underlying neuropathology of PD and various treatments that may impact personality to partially illustrate the need for expertise from multiple disciplines in order to begin to address the complex questions Gilbert and colleagues raise. Finally, we provide some examples of our work to illustrate the importance of interdisciplinary, empirical scholarship based on pragmatism in shedding light on some of the questions Gilbert and colleagues raise.

### Psychological Constructs of Personality and Assessment

Personality psychology has a rich and diverse history. [7] Briefly, personality psychology seeks to understand patterns and variation in how people feel (i.e., affect), behave, and think. [8–12] These variations may be studied at multiple levels. [13–16] Personality has been assessed in several ways in clinical neurology settings. Some measures focus on normal range (non-pathological) personality traits such as the Big Five (i.e., Neuroticism, Extraversion, Openness, Agreeableness, Conscientiousness). [17] Others focus on identifying personality pathology using inventories such as the Personality Assessment Inventory [18] and Minnesota Multiphasic Personality Inventory [19], and yet others assess personality characteristics most often associated with significant damage to regions of the frontal lobes, such as disinhibition and apathy. [20–22] These approaches have advantages such as employing measures with established reliability and validity, yet they have the potential drawback of assessing a relatively limited set of behaviors (i.e., traits, neurobehavioral or neuropsychiatric symptoms) subsumed under the broad domain of personality.

McAdams and Pals [15] articulated one of the most comprehensive multilevel models. Their model encompasses five broad inter-related principles of personality. We focus on three of those principles that are most relevant to clinical neurology settings: dispositional characteristics; characteristic adaptations; and narrative identity.^1^ At the level of basic dispositions, broad personality traits such as the Big Five and their narrower facets or aspects (e.g., assertiveness, enthusiasm) have been defined as relatively uncontextualized, abstract constructs used to either explain or summarize stable, coherent patterns of affect (A), behavior (B), and cognition (C)—the ABCs of personality [23]—over time and space.. Characteristic adaptations are ABC patterns that are contextualized by time, situation, and/or social roles. At the level of characteristic adaptations, people vary on a large number of constructs which comprise motivational (e.g., goals, values) and developmental (e.g., attachment styles) variables, as well as social attitudes (e.g., moral attitudes, prejudice). [16, 17] Finally, the level of narrative identity includes a person’s self-authored life-stories connecting ABC patterns from the past, present, and imagined future into an integrated whole that theoretically provides a person with a sense of unity, coherence, and purpose. Dispositional traits, characteristic adaptations, and integrative life narratives are inter-related and all three comprise the “the social ecology of everyday life”. [15] This model is unique in that it was designed specifically as an integrative way to understand the whole person as opposed to being simply a way of categorizing personality theories or neurobehavioral variables by conceptual similarity. This emphasis is advantageous when applied in the context of DBS, as patients are treated as unique individuals who are more than the sum of defined personality dimensions. Furthermore, the model also incorporates important aspects of pragmatism by explicitly examining the psychological construct of personality through multiple lenses of inquiry while emphasizing the importance of context and the individual’s unique experiential knowledge.

### Personality Changes in PD and DBS through the Lens of Clinicians

In his original monograph describing the disorder named after him, James Parkinson stated that “the senses and intellect being uninjured”. [24, 25] There are now ample data illustrating that Parkinson was incorrect in his assertion. PD is a disorder that can result in significant changes in mood, personality, and cognition either due to the underlying pathological changes associated with the disease and/or treatment side-effects. Although Gilbert and colleagues touch on this literature, it is important to emphasize this point and elaborate on the complexity of assessing personality in the context of PD. First, PD is associated with widespread neuropathological changes impacting multiple neurotransmitter systems, subcortical and cortical regions. [26] The brain regions involved include several that are related to emotion control, motivation, inhibition, judgment, and executive cognitive function . [26, 27] Disruptions in these regions, corresponding neuroanatomical networks, and neurotransmitter systems have been implicated in a host of neuropsychiatric symptoms and disorders. [28, 29] Given the neuropathological changes associated with the disease, it is not surprising that neuropsychiatric symptoms are frequently observed in PD and include dysthymia, depression, anxiety, apathy, hallucinations, and delusions as well as executive cognitive deficits. [30] Some patients may experience increased emotional sensitivity and be more frequently moved to tears (i.e., pseudobulbar-like affect). Further, as the disease progresses, there is greater cortical involvement which can result in increased cognitive and neurobehavioral symptoms which may theoretically impact characteristic patterns of feeling, behaving, and thinking – or the ABCs of personality.

Second, treatments for PD may also result in behavioral changes that might be described as personality changes. For example, dopaminergic medications may result in side effects characterized under the broad term impulse control disorder manifested by hypersexuality, increased spending or gambling, and increased consumption of sweets or salty foods. [31] Other vulnerable patients may experience medication-related hallucinations or delusions. [30, 32] These symptoms typically abate following the reduction or cessation of the medication. Other commonly prescribed PD medications may result in cognitive side-effects, such as poor memory or confusion. Similarly, DBS may result in limbic or cognitive side-effects due to lead placement or stimulation effects. The most common targets for treating motor symptoms of PD (i.e., subthalamic nucleus, globus pallidus interna) are deep nuclei in which there is a specific topography with motor regions located in the dorsal regions adjacent to more ventrally located cognitive and limbic regions. [33–35] The goal with DBS is to stimulate the motor regions of these structures but it is possible that due to lead placement and/or stimulation spread, cognitive and/or limbic regions may be affected resulting in side-effects that may result in neurobehavioral changes suggestive of “personality changes”. In these cases, the stimulation parameters are changed, the stimulator may be turned off or the lead revised and the side-effects disappear.

The critical point with both pharmacological and DBS treatments is that often “personality” changes observed following treatment are not enduring, characteristic changes in patterns of feeling, behaving, or thinking (i.e., the ABCs of personality) and instead reflect *unintended side-effects* of treatment. These side-effects can be addressed by expert DBS teams who have established good communication with the patient and patient’s family. In fact, the ability to titrate stimulation in order to maximize benefits and minimize side-effects is a key advantage of DBS over ablative neurosurgical procedures.

Careful review of some of the narrative data cited as demonstrating personality changes in patients following DBS strongly suggests that at least some of the observed changes may have been related to the underlying disease and/or stimulation side-effects that could be ameliorated with changes in stimulation parameters.

For example, the Patient 07 described by Gilbert and colleagues [36, p99]

“Oh God, I wasn’t me, and I knew I wasn’t me and there was nothing I could do about it … I knew what it was! I knew [DBS] had been turned up that day. Unlike the drugs which creep up on you, and you don’t know what’s happening. With [DBS] I knew what it was, so I knew it was fixable.”

This excerpt clearly indicates that the patient knew that the behavioral change was attributable to the change in stimulation parameters and that the side-effects were “fixable”; the neurobehavioral side-effects of stimulation did not represent enduring changes in personality.

Another excerpt from the same paper highlights Patient 13’s experiences who

“confessed that DBS adversely resulted in intermittent uncontrollable emotional sensitivity, to the extent of experiencing ‘a state of hysterics…I felt like I had lost my true self, it [is] way behind me.’”

Patient 13’s symptoms might reflect a panic attack, increased emotional sensitivity (i.e., similar to pseudobulbar-like affect) or other neuropsychiatric symptoms related to the underlying neurodegenerative changes associated with the disease which were inappropriately attributed to stimulation. Alternatively, if the emotional sensitivity is clearly stimulation related (i.e., abates when the stimulator is turned off and resumes when it is turned on) the behavioral changes may be side-effects that can be addressed by changing the stimulation parameters. It is difficult to ascertain from the quote if these changes were immediately apparent after DBS in a time-locked manner or developed slowly over the course of DBS and ongoing disease progression. The temporal course may provide some indications of the underlying etiology of the behavioral changes which can then provide insight into treating the behavioral symptoms.

In summary, as Gilbert and colleagues [2] noted, the neuropathology of PD as well as the existing empirical literature suggest it is more likely that enduring changes in characteristic patterns of feeling, behaving, and thinking ensue from the underlying neurodegenerative changes associated with the disease rather than DBS. If changes are observed following pharmacological or surgical treatments, they most likely reflect side-effects that can be addressed unless the changes are due to an irreversible surgical complication such as hemorrhage. Finally, it is critical to recall that DBS is not curative; patients will continue to experience ongoing neurodegenerative changes involving regions of the brain which can result in future cognitive and neurobehavioral symptoms that may impact personality. Similarly, the process of living with a neurodegenerative disorder and/or some of the neurobehavioral symptoms that can ensue from treatment (especially impulse control disorder symptoms) may impact interpersonal relationships which may be related to personality. Sorting out the underlying etiology of apparent “personality” changes in patients with PD following DBS depends on expert clinical knowledge, use of empirical methods, and an interdisciplinary approach that specifically incorporates the patients’ and families’ perspectives as well as acknowledgement of the importance of context; all these factors are incorporated in pragmatism. [6, 37, 38] Furthermore, pragmatism calls for a situational lens to understand moral problems because moral agents (their psychology and their behaviors) are embedded in relationships and boast contextual features which can radically shape what is the better option to pursue based on a patient’s values and interests. Likewise, alleged claims of personality changes cannot replace the need of establishing whether these changes occur and what they mean for the patients concerned. Judging from the standpoint of someone outside of a given situation may not do justice to the actual nature of the situation experienced by patients.

### Examples of Empirical Clinical Neuroethics Studies Incorporating Pragmatism

Our team has relied on an interdisciplinary approach to conduct empirical research that specifically elicits patients’ and family members’ perspectives on important questions related to control and personality. Our clinical research team includes clinical ethicists, philosophy trained neuroethicists, personality psychologists, experimental psychologists, statisticians, and clinicians with expertise in neuropsychology, movement disorders and neurosurgery who work daily with patients. As is true in our clinical decision-making, we also intentionally include the voices and experiences of the patients and their family members in our clinical neuroethics research. We have argued before on the need for a variety of different perspectives, skills, and expertise in contributing to best ethical practices in functional neurosurgery teams. [39–41]

- Example 1: Control in the context of DBS for the treatment of motor symptoms of PD.

As Gilbert and colleagues [2] observed, there was a large increase in the number of conceptual neuroethics publications following Gisquet’s seminal paper in 2008. [42] Gisquet argued that DBS results in patients’ loss of control in multiple domains. A fundamental ethical tenet in medicine is to maximize and respect an individual’s retention of bodily sovereignty, inherent in which is the concept of control. We conducted a prospective mixed methods study in patients with PD who underwent DBS exploring different aspects of control including patients’ perceptions regarding control over their symptoms (e.g., reduce tremor, improve gait), control of their behavioral goals (or behavioral motivations to pursue DBS, e.g., continue to work, go to grandson’s football games), and global control of their lives. [43] Participants completed a semi-structured interview in which they identified their top symptom and behavioral goals. Each participant indicated their perceived level of existing control on visual analogue scales (VAS) which provide quantitative measures of control. From a pragmatic standpoint, which starts from the lived experience of patients and other stakeholders, understanding these perspectives is crucial because such perspectives partly constitute the actual situation to tackle. Accordingly, outcomes need to be assessed in a way that reflects the subjective nature of lived experience.

Our data indicated that participants reported significant improvements in control over their individually defined symptom and behavioral goals as well as global life control following DBS. These data suggest that DBS results in positive changes in patients’ lives that are highly individually meaningful and that DBS increases patients’ perceptions of control over their lives. Further, similar to the point Gilbert and colleagues [2] raised, we argued that our findings support a multifactorial assessment of outcome that reflects a diversity of perspectives including the patient, family, and clinical team [40, 43].

We also found that most participants changed the rank order of their symptom and behavioral goals within 3 months following DBS. [44] Whereas changes in rank order were positively correlated with improvements in the symptom goals; the subtler changes in rank order for the behavioral goals were not significantly correlated with improvements in the behavioral goals. This observation suggests that patients’ rank order of symptom goals shift as they experience the benefits of DBS and that once primary symptoms are controlled, other symptoms that are not as well controlled take precedence. Conversely, the behavioral goal data indicate that subtle changes in the participants’ rank ordered behavioral goals were unrelated to the benefits of DBS. In other words, participants still wanted to be engaged with their family, work, and contribute in meaningful ways regardless of whether DBS improved their ability to do so. [44] These data illustrate that the values participants expressed in their behavioral goals did not change directly due to changes associated with DBS; in other words, DBS did not change what participants value most or how they prefer to spend their time.

In the aggregate, these empirical data highlight the important distinctions between different types of control. We acknowledge that the studies were intentionally designed to address the relatively simple concept of control in the domains listed above and not to specifically study conceptual constructs such as PIAAAS. However, our qualitative and quantitative data indicate that DBS significantly improved patients’ perceptions of control in aspects of their lives that are highly individually important (i.e. reflect their values). [43, 44] These findings challenge Gisquet’s [42] thesis that DBS results in a loss of control and suggest that considerations of control may be more nuanced and depend on the frame of reference used (e.g.., control over what, perspective of the individual and/or academic discipline making determinations regarding control). These data highlight the importance of context and different ways of knowing, especially experiential knowledge.

- Example 2: Patients’ and Care Partners’ Perceptions of Personality Change in the context of PD and DBS.

The data from our studies of control illustrated the importance of relationships, avocational pursuits and work (broadly defined) to our patients. Further the data indicated that DBS did not significantly alter patients’ values as reflected in their preferred activities. These empirical observations (coupled with our clinical experiences) led us to question if DBS results in significant changes to personality. Consequently, we developed an empirical methodology to address the relatively narrow question of personality changes (using a psychological definition) in the context of PD and DBS. We are conducting a large mixed methods study that is recruiting participants with PD and their care partners (e.g., spouse, child, friend). Our sample consists of three cohorts: Cohort 1 = patients diagnosed within 1 year; Cohort 2 = patients diagnosed within 5 to 7 years; and Cohort 3 = patients who are approved for DBS. Similar to our previous study, we conduct semi-structured interviews in which patients describe their personality in an open-ended question (similar to probes used to elicit integrative life narratives) followed by participants identifying their top personality characteristics (defined as characteristic patterns of feeling, behaving, and thinking). The subset of participants approved for DBS are prospectively followed over the course of a year to assess changes in individually identified personality characteristics.

Our approach provides an appropriate starting point for gleaning a better understanding of what patients and family members value and how their values are related to existing measures of personality and mood. We recognize that our approach to studying personality may not conform to any existing personality theories or taxonomies but we argue that our emphasis on the patients’ and family members’ most highly valued personality traits is what is most clinically and ethically relevant. Further, these data may help identify those existing personality taxonomies, theories, and measures that best mirror patients’ values and/or may lead to the development of a reliable, valid new personality measure that is highly clinically relevant for patients with brain-based disorders undergoing neuromodulation. Consistent with pragmatist theory, they also suggest that measures need to reflect everyday experiences in a context-sensitive way.

**Fig. 1:**
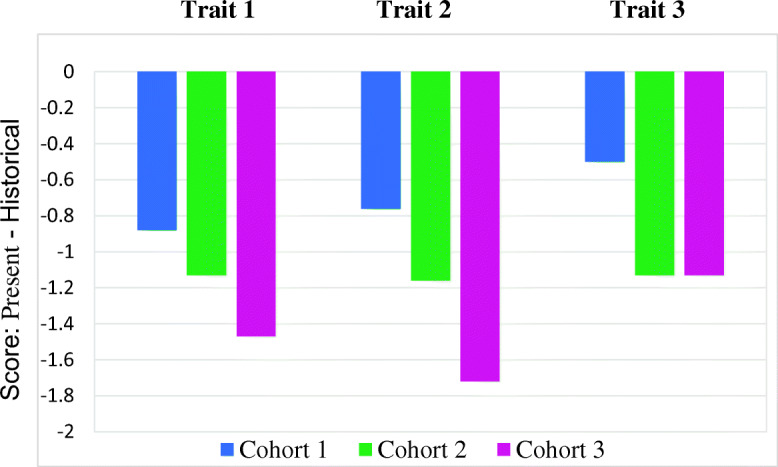
Do patients perceive a change in their most valued personality characteristics over the course of their illness?

**Fig. 2:**
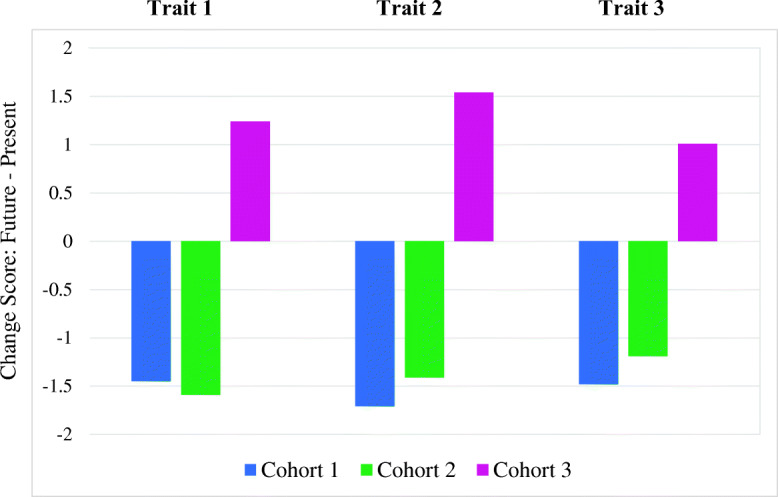
Do patients anticipate future changes in their most valued personality characteristics?

Our preliminary data (based on the initial 25 patients from each cohort) indicate that participants value a diversity of characteristics often embedded in real-world examples more reflective of characteristic adaptations. [45, 46] Most standard clinically used personality measures focus on abstract constructs such as dispositional traits and/or neurobehavioral symptoms and do not comprehensively assess the diverse characteristics our participants cite. [7] Our preliminary analyses demonstrated that participants have observed an average diminution in their individually identified most highly ranked personality characteristics over the course of the disease (based on retrospective ratings) and patients in Cohorts 1 and 2 anticipate even further reductions in the future. In contrast, participants who have been approved for DBS anticipate changes in which their personality traits will be closer to their pre-disease level. [46] (See Figs. 1, 2).

**Fig. 3:**
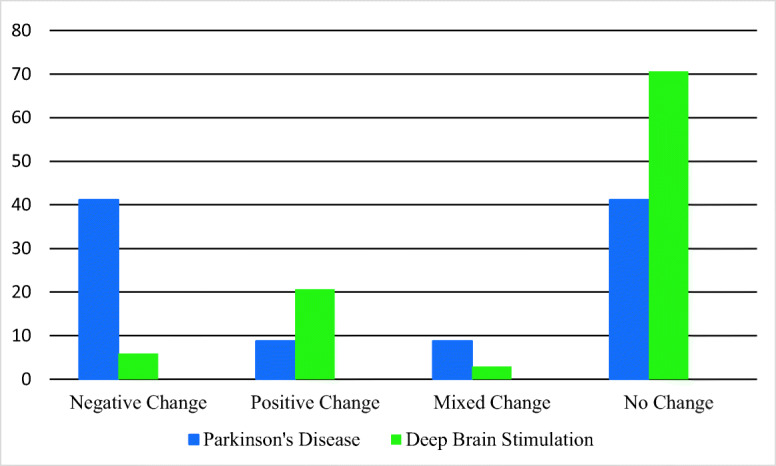
Percentage of patients reporting personality change in the context of Parkinson’s disease vs deep brain stimulation

These early findings mirror some initial pilot data in which a sample of 34 patients were systematically asked to comment on their perceptions of personality change over the course of PD and following DBS as part of a standard clinical interview during pre- and post-operative neuropsychological assessments. [47] These preliminary data revealed that although many patients observed significant personality changes due to PD relatively fewer identified changes following DBS; if changes were identified, PD was associated with more negative changes and DBS was associated with more positive changes (Fig. 3).

These two examples illustrate how conceptual neuroethical questions related to control led to an empirical investigation of different aspects of control in patients with PD over the course of DBS. Some of the findings that emerged from the studies of control then led to conceptual questions regarding the role of DBS in changing fundamental aspects of self which then led to another large, prospective empirical study which focused on the psychological construct of personality. We anticipate that findings from our current study will generate additional conceptual questions. This iterative process of inquiry in which questions raised by the humanities lead to empirical studies which then raise additional conceptual questions is also consistent with pragmatism.

### The Potential Perils of C. P. Snow’s Two Cultures and the Utility of Pragmatism

Gilbert and colleagues [2] clearly documented that several of the conceptual manuscripts they reviewed did not accurately reflect the empirical findings or did not even cite the empirical data. Consequently, the conceptual literature often exaggerated concerns regarding changes in personality in patients who undergo DBS (i.e., the “bubble”). The insufficient acknowledgement of objective data coupled with an incomplete understanding of the complexity of neurobehavioral changes in PD (and other relevant neurological or neuropsychiatric disorders) contributes to a disconnection between the humanities and the sciences which has ethical ramifications. Gilbert and colleagues [2] aptly point out concerns regarding neuroethicists potentially contributing to unfounded fear in patients or the public regarding personality changes following DBS. These fears may inappropriately discourage patients from seeking out potential treatments. In addition, these unsubstantiated claims may inadvertently contribute to negative perceptions of clinician-scientists who offer DBS, and could potentially impact financial support for ongoing neuromodulation empirical trials that may benefit suffering patients. These claims may also hamper important interdisciplinary neuroethics’ scholarship. For example, unsubstantiated claims that DBS results in deleterious changes in personality that do not take into account the complexity of the underlying neurobiology may result in clinicians dismissing the contributions of conceptual neuroethicists in this area since the conceptual claims do not reflect their clinical experience or scientific knowledge (i.e., context). Similarly, some clinician researchers’ single-minded focus on outcome metrics that may not fully capture the patients’ and families’ goals or experiences may contribute to the mis-perception that clinicians are uninterested in or not studying critically important neuroethical questions in DBS. Neuroethics scholarship by its’ very definition is interdisciplinary and should reflect a respect for diverse forms of scholarship. Those who engage in neuroethics’ research need to be mindful of C. P. Snow’s observation; as Kitcher [42, p 40] elaborated, we cannot afford to fall “prey to two forms of short-sightedness, one that embodies attitudes of distrust (or worse) toward the consensus views of natural scientists, and another that dismisses the potential contributions that the humanistic disciplines might take” .

Pragmatism, because of its willingness to bring scientific and experiential knowledge to bear on discussions about ethical matters, can serve as an interesting bridge between divided academic disciplines and discourses, granted that open discussion and the fallible nature of human knowledge be recognized.[48, 49] This process, and fallibilism, certainly require humility, an open-mindedness regarding others’ perspectives, the ability to self-reflect, and an open appreciation of what counts as knowledge. [5] Indeed, instead of quickly opting for reflexes that validate or discount whole domains of human knowledge, pragmatism invites a dialogue between different academic worldviews to deepen our understanding of what we value but also to value what we understand, if knowledge is to guide us in our efforts to resolve difficult moral problems. Within such an interdisciplinary spirit of collaboration, empirical data can contribute to experiential and societal understandings of DBS, inform philosophical discussions, and ultimately improve patient care. At the same time, the worries expressed by humanities scholars should not be dismissed since they often express relevant concerns and questions also shared by members of the public. [50] To move ahead, pragmatism suggests that our concerns and also our solutions be viewed as “hypotheses to test” [51] and that we drop more dogmatic intellectual convictions to yield the way to open deliberation and inquiry and, in this process, make our assumptions more explicit in conceptual bioethics scholarship. [52] It is hoped that more robust knowledge will come from tighter interdisciplinary collaborations where foundational stances will be dropped in favor of the openness to learning from each other, in line with our ultimate objective and aspiration: the growth and flourishing of those we seek to help and understand. [53]

As a result of our empirical research geared toward the bridging of different academic cultures, we submit that research assessing personality (and related concepts) in the context of DBS should be conducted by an interdisciplinary team that includes expert clinicians with intimate knowledge of brain-behavior relationships and extensive experience working with patients who have undergone DBS.[e.g.,^54^] Quite simply, reading about patients with PD who undergo DBS is a very different experience than treating hundreds of patients and families. Inclusion of front-line clinical experts (i.e., neurologists, neurosurgeons, neuropsychologists, psychologists, psychiatrists) helps put the data into the relevant medical, neurobiological, and psychosocial context. As noted above, these patients are very complex and there are multiple variables that can theoretically impact personality. Further, reports published in specialty medical journals often assume that the audience has a clear understanding of the complexity of these patients; thus, the important context is typically not included due to space limitations. Unfortunately, this can potentially contribute to incorrect assumptions by non-clinician readers. Second, patients and family members are a critical source of information to better understand what it is like to live with PD and DBS. Their voices should be included more systematically. This point is particularly important in the context of an elective neurosurgical procedure with the goal of improving quality of life. Finally, pragmatism advocates for the use of empirical methods to help address ethical questions in a way that reflects their situational and experiential dimension. Empirical neuroethics research should be based on data that are collected with a well detailed methodology so that others can replicate those findings. [e.g.,43–47] Ideally, participants should reflect a consecutive series to minimize the possibility of bias in the data. Participants should be very well characterized as should the methods used, including the qualitative methods. Adopting these relatively simple suggestions will result in a more reliable and valid set of empirical data which can inform and enrich conceptual neuroethics scholarship on issues related to PIAAAS in the context of DBS.

## Footnotes

1 The other two principles discussed by McAdams and Pals are: 1) Evolution and Human Nature; and 2) The Differential Role of Culture
